# Current status of radionuclide therapy targeting PARP in cancer: challenges and prospects

**DOI:** 10.1007/s00259-026-08016-9

**Published:** 2026-06-30

**Authors:** Ahmad Kurniawan, Luis Hernandez Cano, Nerea Delgado Mayenco, Sreelakshmi Unnikrishnan, Philip H. Elsinga, Inês F. Antunes, Erik F.J. de Vries, Bart Cornelissen

**Affiliations:** Department of Nuclear Medicine and Molecular Imaging, University Medical Center Groningen, University of Groningen, Hanzeplein 1, Groningen, the Netherlands

**Keywords:** PARP inhibitor, PARP, Targeted radionuclide therapy, Auger electron, Review

## Abstract

In recent years, numerous studies have investigated the use of poly (ADP-ribose) polymerase (PARP) inhibitors, labelled with various radionuclides, as agents for targeted radionuclide therapy. This review discusses current advances in studies with radiolabelled PARP inhibitors for targeted radionuclide therapy, focusing on radionuclide selection and (radio)biological properties. We highlight differences between radionuclides and their efficacy in killing cancer cells, while safeguarding healthy tissue. Furthermore, important biomarkers and dosimetry are explored, providing insights and future directions for advancing radiolabelled PARP inhibitors.

## Introduction

Poly(ADP-ribose) polymerase (PARP) enzymes are a family of structurally similar enzymes that play a critical role in the DNA damage response (DDR), ensuring the maintenance of genomic integrity. In this context, PARP1, also referred to as PARP, is the most numerous and most studied member of the PARP family. In response to DNA damage, PARP1 binds to DNA single-strand breaks, causing an allosteric change in the PARP structure, which activates an NAD^+^ binding pocket and PARP1’s catalytic function: attachment of poly-ADP-ribose (PAR) to itself and other proteins (PARylation). Autoparylation is necessary for PARP1 to detach itself from DNA. PARP inhibitors are small molecules developed to prevent DNA damage repair by engaging the NAD^+^ binding site in the catalytic domain of PARP1. This impairs PARP activity, autoparylation, and therefore causes PARP trapping on the DNA, which eventually leads to accumulation of DNA damage, especially in cells that cannot process the resulting damage, such as those with BRCA1 or 2 mutations (synthetic lethality) [1].

Four PARP inhibitors (olaparib, rucaparib, niraparib, and talazoparib) have been approved for clinical use globally to date, particularly for the treatment of patients with homologous recombination-deficient (HRD) cancers [2–5]. The PARP inhibitor veliparib has not yet been approved by the FDA and is being investigated in combination with chemotherapy in clinical settings [6]. Most recently, the PARP inhibitor saruparib (AZD5305), which is highly selective for PARP1, has entered late stage of clinical trials [7]. Additionally, other PARP inhibitors, such as pamiparib, fluzoparib, and senaparib, have been used in clinical practice in China [8–10].

As PARP inhibitors bind to PARP in cancer cells, which exhibit higher PARP expression due to genomic instability, and PARP is associated more with tumour DNA, the inhibitors can be used as vectors for radionuclide delivery, enabling applications in both imaging and targeted radionuclide therapy (TRT). Unlike conventional PARP inhibitors, PARP-TRT induces direct cytotoxic DNA damage that may partially bypass dependence on HRD. Importantly, therefore, this radiation-mediated cytotoxicity may also retain efficacy in tumours that have developed resistance to conventional PARP inhibitors, such as through HR restoration [11]. As a result, PARP-TRT has the potential to target a far broader range of cancer cells, not limited to those with BRCA mutations [12, 13].

Several radiolabelled PARP inhibitors have been developed as molecular imaging agents intended for detecting cancer, monitoring PARP expression, and pharmacokinetic/pharmacodynamic imaging to improve PARP inhibitor efficacy. Some of these have been evaluated in cancer patients for imaging purposes and as therapeutic biomarkers, including [^18^F]FluorThanatrace ([^18^F]FTT) in ovarian and breast cancer patients [14, 15] and [^18^F]PARPi in head and neck cancer patients [16].

**Fig. 1:**
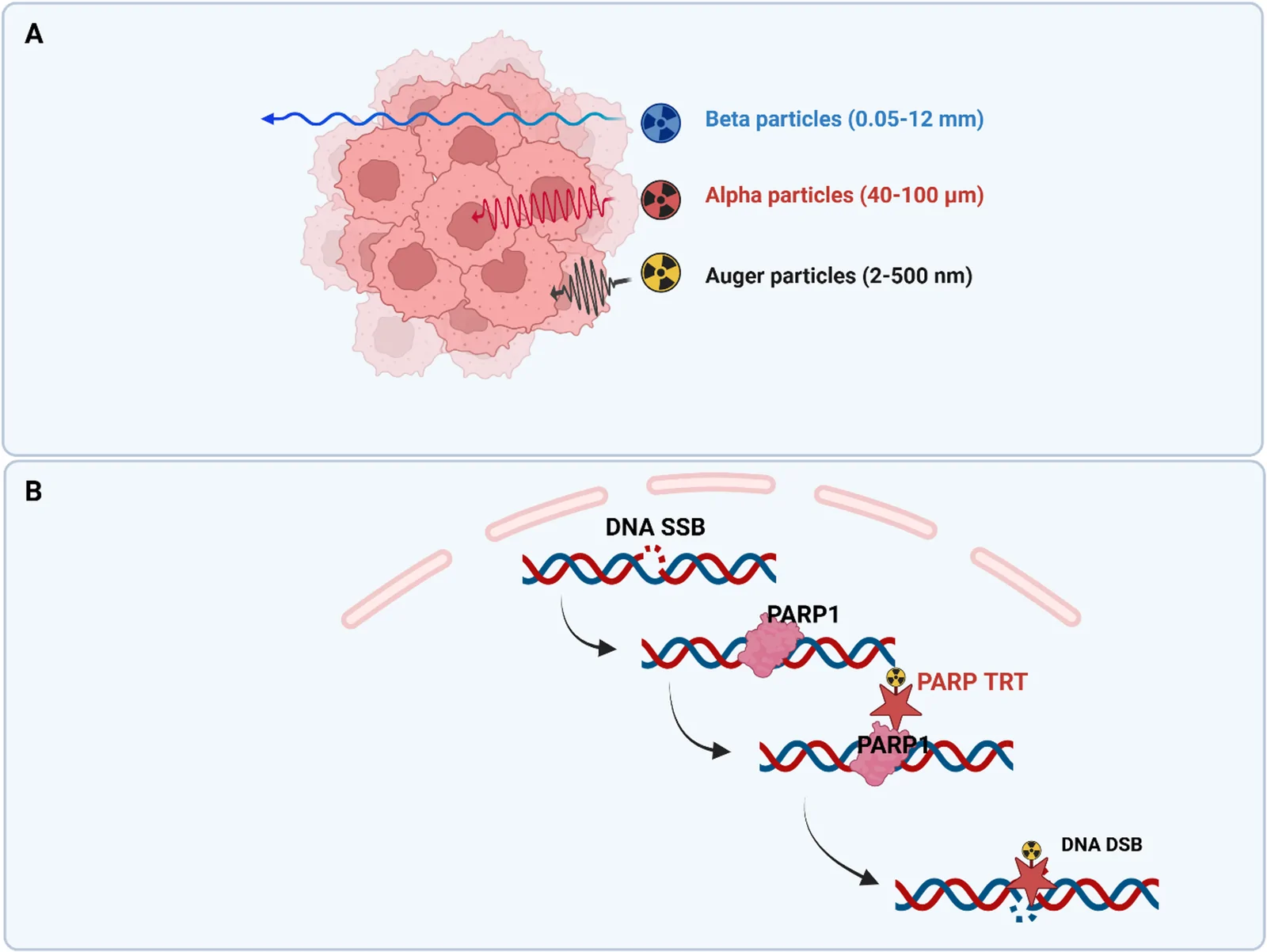
Targeted radionuclide therapy with radiolabelled PARP inhibitors in cancer. Radionuclides of various types, including alpha, beta, and Auger electron emitters, with distinct energies and penetration depths, are used in targeted radionuclide therapy to selectively kill cancer cells (**A**). Radiolabelled PARP inhibitors bind to PARP enzymes that are engaged with unstable DNA in cancer cells, inducing double-strand breaks that lead to cancer cell death (**B**). (Image generated using Biorender)

Radiopharmaceuticals also hold immense potential as anticancer therapeutics, as they can be used to kill cancer cells by precisely delivering radiation to the tumour, while avoiding damage to healthy tissue. Therapeutic radiopharmaceuticals usually consist of cancer cell-targeting small molecules, peptides, proteins, or particles labelled with alpha-, beta-, or Auger electron-emitting radionuclides (Fig. 1). Alpha-particles are densely ionising, with a high LET of 80–100 keV/µm, and a short range (approx. 30 μm in tissue). Alpha particle-emitting radionuclides have been employed in TRT, e.g. ^225^Ac, ^211^At, and ^212^Bi. In contrast, beta particles are characterised by their deeper tissue penetration (several mm), yet lower LET of approximately 0.2 keV/µm. Radiopharmaceuticals labelled with a beta particle-emitting radionuclide are commonly used for bulk tumour treatment. Radiopharmaceuticals labelled with ^177^Lu and ^161^Tb are prominent examples. Auger electrons (AE) have a relatively high LET of 4–26 keV/µm, with a very short tissue range (several nm). As AEs are characterised by their ultra-short range and highly localised energy deposition at the nanometre scale, they cause minimal effects in non-targeted cells but induce significant damage in targeted tumour cells. In particular, their high LET leads to densely clustered DNA damage, especially when Auger electrons are in close proximity to DNA [17–19].

Accordingly, radiolabelled PARP inhibitors with radionuclides emitting alpha particles (^211^At [20–24]), beta particles (^177^Lu [25], ^131^I [26, 27]), as well as Auger electrons (^125^I [12, 13, 28–32], ^123^I [33–39], ^77^Br [40–42]) have been previously investigated and are at various stages of development at the time of writing. The selection of the radiolabelled PARP inhibitor for TRT should involve a careful consideration of the physical properties of the radionuclide, including particle type, half-life and suitability for therapy and imaging. A summary of the most important properties, advantages and disadvantages of the radionuclides used to label PARP inhibitors is provided in Table 1.

This review highlights recent advances in the development of radiolabelled PARP inhibitors for cancer therapy, with an emphasis on radionuclide selection and radiobiological considerations.

**Table 1:** Properties of radionuclides used with PARP-targeting radiopharmaceuticals for therapy

Radionuclides	Half-life	Emitted particles	Advantages	Disadvantages	References
Astatine-211 (^211^At)	7.2 h	6.79 MeV alpha particles	− 100% single α emitter, which prevents unpredictable dose localisation from daughter radionuclides through decay - α particle emissions deposit large amounts of energy over distances of 50–70 μm (high LET) - Short half-life matching the rapid kinetics of most small molecule and peptide-based radiopharmaceuticals - Radiohalogen chemistry, but only partially characterised - Decays into Polonium-211, which has an ultra-short half-life (highly localised tumour killing) - Emits 77–92 keV X-rays, allowing SPECT imaging	- Production facility cost and availability (need for a high-energy cyclotron) - Distribution limitation due to its short half-life - No stable isotope of astatine to use as a reference standard - Stability issues in vivo may lead to deastatination - The radiochemistry of At-211 remains challenging and less established, compared with other radionuclides	[22, 43–45]
Iodine-131 (^131^I)	8.02 d	192 keV β-particles	- Emits high-energy β particles to target the bulk of the tumour - Established radiohalogen chemistry	- Poor image quality due to high photon energy - No match between highly precise targeting (for targeting PARP) and long beta particle range	[26, 27, 46]
Lutetium-177 (^177^Lu)	6.7 d	134 keV β-particles	- Emits high-energy β particles to target the bulk of the tumour - Energy γ 208 keV allows for SPECT imaging - Established radiometal chemistry	- Radiometal chemistry requires a chelator that reduces lipophilicity (limited membrane penetration) - No match between highly precise targeting (for targeting PARP) and long beta particle range	[25]
Iodine-123 (^123^I)	13.3 h	13.7 Auger electrons/decay	- Enables SPECT imaging - High LET due to significant Auger electron emission/decay - Established radiohalogen chemistry - Wide availability	- Short range of Auger electrons, which necessitates highly precise targeted delivery to tumour DNA	[36, 38, 47]
Bromine-77 (^77^Br)	57 h	6.6 Auger electrons/decay	- Established radiohalogen chemistry - Theranostic pair with ^76^Br as a positron emitter for PET imaging	- Limited availability due to production challenges (cyclotron capabilities and purification) -Short range of the Auger electron, which needed precise targeted delivery	[40, 41], 47– [49]
Iodine-125 (^125^I)	59.4 d	23 Auger electrons/decay	- High LET due to significant Auger electron emission/decay - Commercially available	-Long half-life (causing logistical issues with radioactive waste) - Low molar activity (challenging to achieve high molar activity for labelling) - Short range of the Auger electron, which needed precise targeted delivery	[13, 30, 31]

### PARP inhibitors labelled with alpha-emitting radionuclides

#### [^211^At]Parthanatine

Makvandi et al. investigated the radiolabelled rucaparib-like PARP inhibitor derivative parthanatine with [^211^At] (also known as [^211^At]MM4 or [^211^At]PTT) (Fig. 2.4). In vitro, the compound demonstrated high affinity for PARP1 and markedly increased cytotoxicity compared with free Na[^211^At]At and unlabelled KX1 with added Na[^211^At]At as a non-target control. Mechanistically, exposure to [^211^At]Parthanatine induces DNA damage, as evidenced by an increased number of γH2AX foci, accompanied by elevated PARP1 expression and caused cell cycle arrest at the G2/M checkpoint [22].

**Fig. 2:**
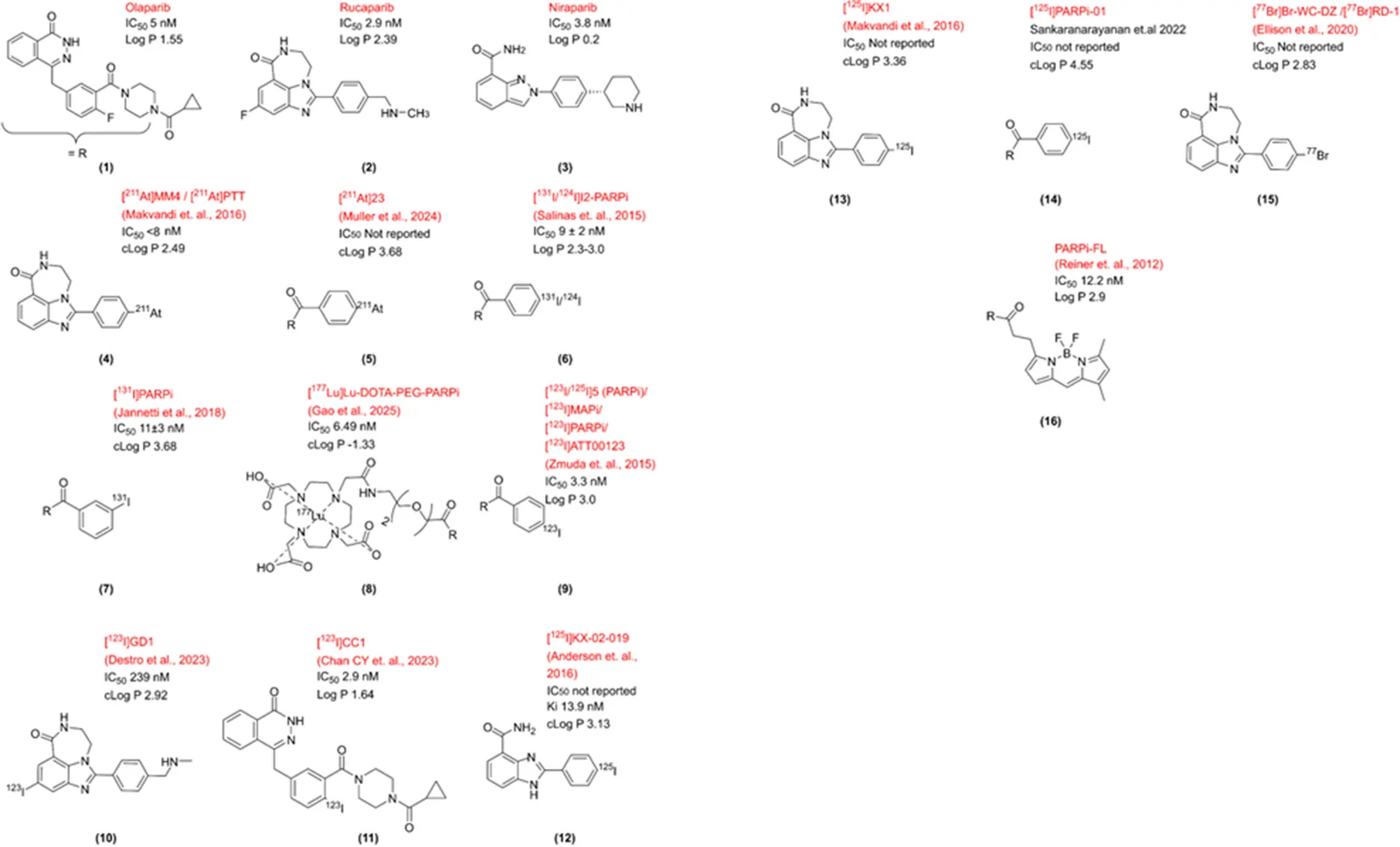
PARP inhibitor structures that have been used as ligands for PARP-targeted radionuclide therapy

In vivo studies in neuroblastoma and glioblastoma models showed blockable tumour uptake and significant antitumour efficacy. A fractionated dosing regimen achieved tumour growth inhibition with reduced toxicity compared to a single high-dose administration [22, 24]. In preclinical patient-derived xenograft (PDX) and syngeneic mouse models, [^211^At]Parthanatine showed antitumor activity at a maximum tolerated dose (MTD) of 36 MBq/kg/fraction (intraperitoneally twice weekly over four fractions) with a higher MTD in immunocompetent C57BL/6 mice of 48 MBq/kg/fraction. Although dose-dependent lymphocyte depletion was observed after the final dose fraction, the neutrophil counts were unaffected due to their low PARP1 expression [21]. In a GL26 subcutaneous mouse glioblastoma model, combining [^211^At]Parthanatine with an anti-PD-1 antibody significantly enhanced tumour growth inhibition and reduced tumour burden, although this was followed by eventual tumour progression [20].

The compound also shows potential for combination with anti-PD1 immune checkpoint inhibitor in a syngeneic glioblastoma model, which may further enhance therapeutic outcomes. Although these results are promising, they require further validation in other tumour types. Additionally, long-term toxicity assessments are lacking, and studies in tumours with varying levels of PARP expression are needed to fully evaluate its therapeutic potential.

#### [^211^At]23

Muller et al. radiolabelled an olaparib-like compound with ^211^At via ^211^At-astatodegermylation (Fig. 2.5). The biological evaluation of [^211^At]23 has not been reported to date [43]. This study focused only on the development of a novel labelling method using astatine-211. Exploring [^211^At]23 further is of particular interest, as recent studies have demonstrated that delivering α-particles in close proximity to DNA results in greater cytotoxic effects [50].

### PARP inhibitors labelled with beta-emitting radionuclides

#### Para-[^131^I]PARPi

Salinas et al. developed p-[^131^I]PARPi (Fig. 2.6) as a SPECT imaging agent that differs from other compounds by placement of the iodine moiety in the para, rather than the meta, position. In vitro, the compound showed high affinity for PARP1 and nuclear binding in U87MG cells, providing indirect evidence of nuclear localisation, confirmed by competition studies using a fluorescently tagged PARP1 inhibitor (PARPi-FL; Fig. 2.16) or by co-incubation with p-iodo-PARPi. In vivo and ex vivo evaluations demonstrated tumour uptake in the U251MG subcutaneous xenograft model, with higher accumulation compared to the normal brain. Tracer specificity was confirmed by a blocking study using the PARP inhibitor olaparib [27, 51].

Overall, p-[^131^I]PARPi showed specific PARP1-mediated tumour uptake and potential utility for SPECT imaging of gliomas in vivo. Although p-[¹³¹I]PARPi demonstrated specific PARP1 targeting in vitro and in vivo, clearer evidence is needed to confirm tumour blocking in vivo. The therapeutic potential of this compound remains unknown, as it has not yet been investigated.

#### Meta-[^131^I]PARPi

Jannetti et al. evaluated an iodine-131 labelled olaparib analogue (m-[^131^I]PARPi) (Fig. 2.7) as a therapeutic agent for glioblastoma. In vitro, the compound showed specific uptake in U251MG cells, with reduced uptake upon blocking with an excess of the PARP inhibitor olaparib. In subcutaneous U251MG xenografts and orthotopic U87-p53 glioblastoma mouse models, SPECT imaging and autoradiography confirmed tumour retention and PARP-specific targeting. Intratumoral administration was performed to achieve a favourable distribution within the target tissue. Administration of m-[^131^I]PARPi induced an increase in p53Luc signal, indicating enhanced DNA damage–driven p53 activation, which may lead to cell death [26]. Repeated dosing via intratumoral administration resulted in reduced tumour growth and prolonged survival compared to vehicle or non-radioactive m-PARPi. Notably, convection-enhanced intratumoural delivery (CED) improved intratumoral distribution and increased the radiation dose to the tumour relative to normal tissue [26].

Taken together, the in vitro, in vivo, and autoradiography results demonstrate that m-[^131^I]PARPi specifically targets the tumour and delivers a therapeutic radiation dose. However, intratumoral injection might be necessary to achieve a higher radiation-absorbed dose to the tumour. The data published so far provide proof of concept for m-[^131^I]PARPi as a beta-emitting agent for PARP-TRT. The compound also shows potential for combination therapy with the immune checkpoint inhibitor anti-PD1 in a syngeneic glioblastoma model, which may further enhance therapeutic outcomes. Although these results are promising, they require further validation in other tumour types. Additionally, long-term toxicity assessments are lacking, and studies in tumours with varying levels of PARP expression are needed to fully evaluate its therapeutic potential.

#### [^177^Lu]Lu-DOTA-PEG-PARPi

While most PARP-TRTs are based on radiohalogens, Gao et al. developed a Lutetium-177-labelled PARP inhibitor, [¹⁷⁷Lu]Lu-DOTA-PEG-PARPi (Fig. 2.8), incorporating a PEG linker to improve solubility and pharmacokinetics, with a goal to reduce liver and intestinal uptake. In vitro, PEGylation increased cellular association compared to the non-PEGylated analogue; however, target-specific binding, blocking, or cellular internalisation were not assessed. In vivo imaging demonstrated tumour signal, and treatment produced a dose-dependent but modest inhibition of tumour growth with 30 MBq of administered activity [25].

Overall, these results indicate that PEGylation substantially improves tumour targeting and pharmacokinetics of [^177^Lu]Lu-DOTA-olaparib. However, the compound’s specificity in vitro or vivo has not been investigated, and a relatively high administered activity (30 MBq) is required to achieve a modest therapeutic response in the study. This warrants further investigation.

### PARPi labelled with Auger electron-emitting radionuclides

#### [^123^I]GD1

Destro et al. developed the radioiodinated rucaparib analogue, GD1 (Fig. 2.10). Although GD1 retained some PARP1 binding capability, its affinity was substantially lower than that of rucaparib. In vitro, [^123^I]GD1 showed rapid cellular uptake with limited retention, and partial inhibition of uptake was observed upon PARP blockade, indicating limited target specificity. [^123^I]GD1 exhibited substantial cytotoxicity in PARP1-expressing cell lines. In vivo, the [^123^I]GD1 was known to be predominantly cleared via hepatobiliary and renal pathways [34].

Overall, the reported data suggest that [^123^I]GD1 may have limited potential as a theranostic agent, as it shows some amount of specific binding to PARP, resulting in a reduction in clonogenic survival in high-PARP1-expressing cancer cells. However, its relatively weak PARP1 binding affinity limits its potential, and in vivo antitumor uptake, efficacy, and toxicity have not yet been demonstrated in the study, which warrants further investigation.

#### [^123^I]CC1

Chan et al. developed [^123^I]CC1, an iodine-123 labelled olaparib analogue (Fig. 2.11), which demonstrated high PARP inhibitory potency and specific uptake across multiple cancer cell lines. In vitro, treatment with [^123^I]CC1 induced significant DNA damage and reduced clonogenic survival across different cell lines. In vivo studies showed tumour uptake and significant tumour growth inhibition. Notably, differences in treatment response were observed across tumour types that could be explained by PARP1 expression levels. Minimal uptake and a lack of efficacy were observed in MDA-MB-231 tumour. [^123^I]CC1 showed no detectable toxicity in normal tissue [39].

Overall, [^123^I]CC1 exhibits potent PARP-targeted cytotoxicity, effective tumour-selective accumulation, and promising theranostic potential that combines imaging and radiotherapy, though cellular uptake and therapeutic response are influenced by factors beyond PARP expression alone and warrant further investigation.

#### [^125^I]KX-02-019

Anderson et al. developed [¹²⁵I]KX-02-019 (Fig. 2.12), an iodine-125-labelled niraparib analogue, as a proof-of-concept agent for imaging PARP expression. The compound showed moderate PARP inhibitory potency and, notably, a preference for PARP2 over PARP1 based on enzyme specificity studies. In vivo, [¹²⁵I]KX-02-019 demonstrated tumour uptake with a favourable tumour-to-background ratio and hepatobiliary clearance, consistent with other PARP inhibitors. However, a relatively rapid washout from the tumour was observed. Importantly, partial in vivo deiodination led to off-target thyroid accumulation, raising concerns about tracer stability [31]. Its potential use in PARP-TRT was not explored in the study.

#### [^123/125^I]KX1

Makvandi et al. evaluated an iodine-125-labelled rucaparib analogue, KX1, as a proof-of-concept agent for PARP1 imaging and targeted radionuclide therapy (Fig. 2.13). The compound exhibited high binding affinity comparable to olaparib and demonstrated PARP1-specific uptake in vitro, supported by knockout models and inhibition studies, alongside dose-dependent increases in DNA damage [12, 22, 28, 29].

In contrast, in vivo study showed no significant difference in tumour uptake between control and olaparib-pretreated animals. Ex vivo analysis showed that blocking with veliparib significantly reduced uptake, indicating target specificity [12, 32].

To date, the evaluation of iodine-123–labelled KX1 has been limited to radiolabelling optimisation and assessment of its in vivo imaging capability as a PARP1-specific radiotracer [32]. However, in vivo studies did not yield very promising results, as no blocking effect was observed. No studies have yet been reported evaluating its therapeutic efficacy as an Auger electron-emitting therapeutic agent.

#### Para-[^125^I]PARPi

p-[^125^I]PARPi was evaluated as a potential Auger electron emitting radiopharmaceutical for PARP-TRT in triple-negative breast cancer (Fig. 2.14). In vitro, the compound showed PARP-specific uptake and induced significant DNA damage, leading to a reduction in colony survival, apoptosis, and G2/M cell-cycle arrest, all potentiated by pre-treatment with liposomal doxorubicin [30]. However, an in vivo study of p-[^125^I]PARPi showed no significant survival benefit, despite a significant increase in tumour apoptosis compared with vehicle-treated controls [13].

Poor tumour accumulation and high off-target uptake in vivo may limit the therapeutic benefit of the compound. These findings support the concept of PARP-targeted Auger therapy but warrant further improvement for clinical translation.

#### [^77^Br]Br-WC-DZ

Hoffman et al. evaluated [^77^Br]RD1, also referred to as [^77^Br]Br-WC-DZ (Fig. 2.15), a rucaparib-based PARP-TRT. Radiotoxicity was higher in PARP1-expressing OVCAR8 cells compared with PARP1-knockout controls, consistent with reduced binding capacity in the knockout model and lower EC_50_ values in PARP1-positive cells [41, 42]. Similarly, Sreekumar et al. reported strong antiproliferative effects of [⁷⁷Br]Br-WC-DZ in prostate cancer cell lines, with markedly higher potency than the non-radioactive analogue. The compound induced dose-dependent cytotoxicity, as evidenced by DNA damage, G2/M cell cycle arrest, and increased markers of DNA double-strand breaks [40].

In vivo, [^77^Br]Br-WC-DZ showed tumour uptake in a prostate cancer xenograft model, which was significantly reduced by co-administration of excess olaparib, confirming PARP-specific targeting. The therapeutic evaluation of [⁷⁷Br]Br-WC-DZ significantly prolonged survival with minimal toxicity [40].

These results support the use of [⁷⁷Br]Br-WC-DZ as a potent, mechanism-driven candidate for PARP-TRT.

### PARP-targeted radionuclide therapy in the clinic

**Fig. 3:**
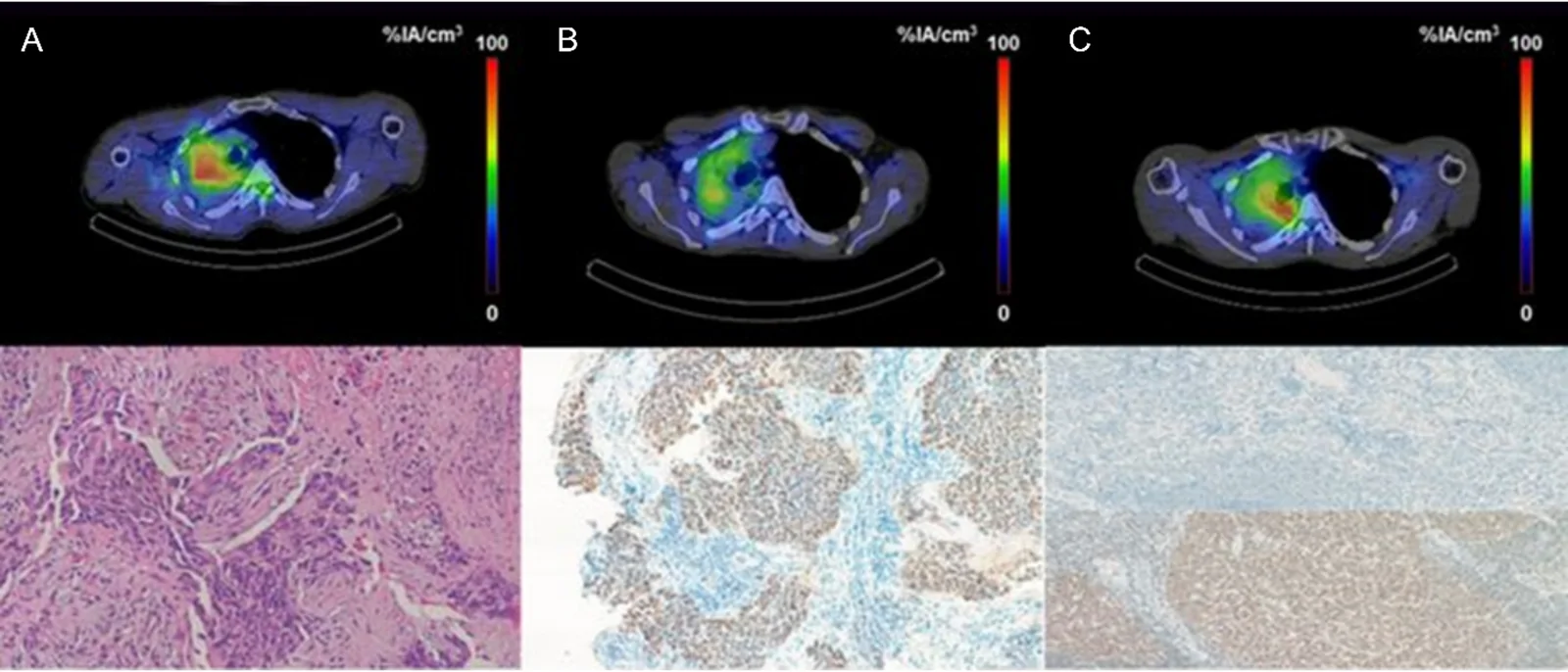
SPECT/CT image of p-[ ^123^ I]PARPi. p-[^123^I]PARPi was administered intravenously to a patient with a right-sided small cell neuroendocrine carcinoma who was receiving carboplatin and etoposide. Panel A shows baseline imaging, Panel B signifies early response assessment (two cycles of carboplatin and etoposide), and Panel C presents the final therapy (six cycles of carboplatin and etoposide). The baseline image presents a hypermetabolic lung mass, while immunohistochemistry staining from the biopsy sample shows PARP expression on panels B and C. Reproduced with permission from Ndlovu et al. [52]

Numerous preclinical studies have investigated PARP-TRT candidates; however, no completed clinical studies have yet been reported. A first-in-human study evaluated p-[^123^I]PARPi as an imaging agent to assess PARP upregulation in a patient with small-cell neuroendocrine carcinoma of the right lung. Administration of p-[^123^I]PARPi resulted in uptake within a pulmonary lesion in a patient receiving carboplatin and etoposide, and immunohistochemical staining confirmed high PARP expression in the corresponding tissue (Fig. 3) [52].

A phase I clinical trial has been conducted to evaluate p-[^123^I]PARPi (also known as [^123^I]ATT001; Ariceum Therapeutics). In this study (NCT06650605), the agent is administered as monotherapy via intracavitary direct instillation and in combination with other anticancer therapies in patients with relapsed glioblastoma. The primary objectives are to assess the safety and tolerability of p-[^123^I]PARPi and to determine the maximum tolerated dose. A total of 7 patients were enrolled, and the reported study completion date was 1 March 2026 [53].

#### Para-[^123^I]PARPi

Zmuda et al. investigated the PARP-targeted Auger electron-emitting p-[^123^I]PARPi also referred to as [^123^I]MAPi or [^123^I]ATT00123 (Fig. 2.9). The compound exhibited high PARP1 binding affinity and PARP-specific uptake as confirmed by olaparib blocking studies [35]. An in vitro study by Pirovano et al. also showed rapid cellular internalisation and specific uptake, including nuclear and chromatin localisation, in glioblastoma (U251) and small cell lung cancer (LX22) cells. The compound also demonstrated greater cytotoxic potency compared to p-[^131^I]PARPi, whereas unlabelled olaparib had no measurable effect at the same concentration. Substantial DNA damage [36, 38]and reduced clonogenic survival were also observed [36, 38].

An in vivo study demonstrated high, yet highly variable tumour uptake, which was markedly reduced by co-administration of excess olaparib, confirming target-specific binding. A therapeutic study in a glioblastoma mouse model showed prolonged survival following intratumoral administration or CED. Additionally, the feasibility of delivering p-[^123^I]PARPi across the blood–brain barrier was evaluated via intrathecal injection, enabling targeted delivery to brain tumours [38].

Wilson et al. evaluated the antitumour effect of systemically administered p-[¹²³I]PARPi in colorectal cancer xenografts with differing p53 status. Tumours lacking functional p53, which impairs DNA damage repair, showed higher tracer uptake and increased DNA damage compared to p53 wild-type models. While no significant tumour growth inhibition was observed in p53 wild-type tumours, treatment led to a marked improvement in survival in p53-deficient models. These findings suggest that p53 status may influence response to Auger electron-emitting PARP-TRT and could serve as a potential determinant of therapeutic efficacy. Furthermore, a toxicity study in healthy female athymic nude mice following intravenous administration of 74 MBq of p-[^123^I]PARPi showed only transient increases in liver toxicity biomarkers, which returned to normal levels after two weeks [37].

The Auger electron-emitting compound p-[^123^I]PARPi is a promising PARP-TRT that demonstrates high PARP1 binding specificity, efficient tumour uptake, and potent DNA-damaging effects in vitro and in vivo. Moreover, p-[^123^I]PARPi showed rapid cellular internalisation, nuclear localisation, and strong clonogenic inhibition, resulting in superior relative biological effectiveness compared to conventional external beam radiation. Preclinical studies across glioblastoma, small-cell lung cancer, and colorectal cancer models indicate that p-[^123^I]PARPi can prolong survival with minimal toxicity, highlighting its potential as a targeted therapeutic for PARP1-expressing tumours.

## Discussion

### Mechanistic insight into PARP-Targeted approaches

PARP inhibitors have been used in different applications, including as a monotherapy, radiosensitisation for external beam radiotherapy (EBRT), and as a targeting vector for TRT. A better understanding of the mechanisms of action underlying each application will provide further insight for future studies. As monotherapy, PARP inhibitors prevent PARylation by inhibiting the catalytic activity of PARP, thereby impairing the repair of DNA single-strand breaks. In homologous recombination (HR)-deficient cells, this leads to replication fork collapse and subsequent accumulation of DNA double-strand breaks, a process known as synthetic lethality [1].

Although used as monotherapy, PARP inhibitors have also been extensively investigated as radiosensitizers, with the aim of enhancing the efficacy of EBRT while reducing toxic side effects to surrounding normal tissues. Evidence also suggests that loss or inhibition of PARP-1 and PARP-2, both of which are activated in response to DNA damage and facilitate DNA repair, leads to marked hypersensitivity to ionising radiation. Accordingly, inhibition of PARP-mediated DNA repair prolongs DNA strand breaks and promotes the activation of cell death pathways, thereby increasing radiation-induced cytotoxicity [54].

Meanwhile, PARP-TRT combines PARP’s DNA damage-sensing function with radionuclide delivery using a PARP inhibitor as a targeting vector linked to radionuclides. This approach enables radionuclide accumulation at sites of DNA damage and within the nucleus, where PARP is predominantly localised. The emitted particles (Auger electrons, beta particles, or alpha particles) are intended to induce highly localised DNA damage, increasing double-strand breaks and cytotoxicity in tumour cells, as already mentioned in previous sections. Overall, this strategy exploits PARP-mediated nuclear targeting to concentrate ionising emissions at chromatin, thereby enhancing tumour cell kill while sparing normal tissues.

### Radionuclide selection and ligand design for PARP-TRT

The selection of an appropriate radionuclide remains a critical decision in the development of PARP-TRT. Each radionuclide offers distinct advantages and limitations, as summarised in Table 1. Given that PARP is located within the nucleus of cancer cells [55], radionuclides capable of delivering high-LET radiation over subcellular distances are preferred for PARP-TRT, as opposed to longer-range beta emitters. Auger electron emitters, characterised by very short path lengths and high LET, are particularly well suited to induce clustered, complex DNA damage when localised inside the nucleus, adjacent to DNA. Several Auger electron-emitting radionuclides, including ^123^I [38, 39], ^125^I [12, 31], and ^77^Br [40, 41], are being investigated for PARP-TRT applications.

The use of Auger electron emitters is critical to ensure that PARP-TRT selectively kills cancer cells with high PARP expression while sparing surrounding normal tissue. This strategy may be particularly advantageous for the treatment of smaller tumour clusters or micro metastases, which frequently recur after conventional cancer therapy. In contrast, the use of β- or α-emitting radionuclides for PARP-TRT poses dissonances with nuclear-DNA-specific targeting. Limitations such as the crossfire effect may increase collateral damage to adjacent tissues, especially in tissues, such as the gallbladder and liver, inherently involved in the excretion of radiopharmaceuticals with sufficient lipophilicity to allow them to cross tumour cell membranes, necessary for reaching nuclear PARP [56]. In addition to the previously mentioned PARP-targeted imaging agents labelled with radiohalogens [14–16, 26, 27, 38, 39], several radiometals labelled compounds, such as those incorporating ^64^Cu [57], ^68^Ga [58–60] and ^99m^Tc [61], have also been investigated for PARP imaging. Although target-specific binding has been demonstrated for several radiometal labelled PARP tracers, the extent of blocking, cellular uptake, and tumour accumulation is generally lower than reported for many radiohalogenated PARP tracers. This finding suggests that incorporating a radiometal–chelator complex may adversely affect the physicochemical properties required for efficient intracellular uptake and subsequent engagement of nuclear PARP.

The clinical application of β-emitting radiometals, such as ^177^Lu, as well as the promising characteristics of ^161^Tb, which also emits Auger electrons, has been explored for TRT using vectors including DOTATATE, LM3, PSMA, or FAPi [62]. However, the targets of these vectors are all located on the outer cell membrane. Whether these radiometals offer potential for further development as PARP-TRT candidates remains to be explored. Although alpha-emitting radiometals such as ^225^Ac, ^149^Tb, ^212^Pb, or ^212^Bi are also compelling candidates for similar applications, diffusion across the cell membrane may be significantly hindered by the hydrophilic nature of the radiometal-chelator moiety [63].

PARP-TRT ligands are typically designed based on the structural scaffolds of clinically established PARP inhibitors such as olaparib, rucaparib, and niraparib. In the case of olaparib-derived compounds, the phthalazinone core, coupled with a piperazine–benzamide moiety is critical for binding to the NAD⁺ binding pocket of PARP1/2, thereby enabling selective delivery of radionuclides to the target [64, 65]. Structural modifications are generally introduced with the aim of preserving lipophilicity, membrane permeability, and high binding affinity to the PARP enzyme, as consistently demonstrated across most of the reported compounds (Table 2).

**Table 2:** Summary of currently reported PARP-targeted radionuclide therapies

#Compound	Cancer types and cell lines	IC_50_/EC_50_		In vitro biomarker (2D)	In vivo dose, route and therapeutic effect	Reference
		Cell-free	In vitro (cell line)			
4. [^211^At]MM4 / [^211^At]PTT MA = 950.8-16021 GBq/µmol	Neuroblastoma (18 cell lines), Ovarian (5 cell lines), breast (7 cell lines) and glioblastoma (4 cell lines)	KX1 ≤ 8 nM	≈ 0.026 kBq/mL (IMR-05), ≈ 5.5 kBq/mL (NLF)	γH2AX foci increase; G2/M arrest; Ki-67 reduction	4 × 370 kBq (i.v., twice weekly); tumour regression with no regrowth up to 80 days; Target blocking confirmed	[20–24]
5. [^211^At]23 MA = ND	ND	ND	ND	ND	ND	[43]
6. Meta-[^131^I]PARPi/[^131^I]PARPi MA = 1.5 ± 0.3 GBq/µmol	Glioblastoma (U251MG and U87-p53/tdTomato-CBRluc-Neo cells )	11 ± 3 nM	ND	p53 increase	3 × 14.8 MBq (intratumoral): reduce tumour volume with prolonged median survival (29 days); 1 × 30.7 MBq via CED: higher radiation absorbed dose to tumour; Target blocking confirmed	[26]
7. Para-[^131^I]PARPi/[^131^I/^124^I]I2-PARPi MA = ND	Glioblastoma (U251MG and U87MG)	9 ± 2 nM	ND	ND	SPECT imaging only	[27]
8. [^177^Lu]Lu-DOTA-PEG-PARPi MA = 3.7 GBq/µmol	A549, AsPC1, ISN-1, and U87MG	Not reported	9.81 nM	ND	1 × 10, 20, or 30 MBq (i.v.); 10 and 20 MBq; low inhibition at 10 and 20 MBq; moderate tumour growth inhibition at 30 MBq; Target blocking not confirmed	[25]
9. Para-[^123^I]PARPi/[^123I/125^I]5 (PARPi)/[^123^I]MAPi/ [^123^I]PARPi/ [^123^I]ATT001 MA = > 10 GBq/µmol	Glioblastoma (U251, TS543, G7 and T98G), colorectal cancer (HCT16 p53^+/+^, HCT16 p53^−/−^), FaDU (Hypopharyngeal squamous cell carcinoma), and Glioblastoma (U251)	69 nM	7.0 (6.3–7.9) nM for G7 cells; 7.4 (6.4–8.5 nM) for T98G cells; 11 nmol/L (HCT16 p53^+/+^), and 22 nmol/L (HCT16 p53^−/−^)	γH2AX increase; elevated apoptosis (TUNEL); p21 increase	1 × 0.37–1.11 MBq (intratumoral): improved survival up to 58 days; 481 ± 111 kBq via CED over 5 days, prolonged median survival (72 days) 1 × 74 MBq (i.v.): no significant tumour growth delay; Target blocking confirmed Phase1 Clinical Trial ([^123^I]ATT001 NCT06650605) in patients with relapsed glioblastoma	[35–38, 52]
10. [^123^I]GD1 MA = 0.02 GBq/ µmol	Pancreatic cancer (AsPC1, PSN1) and Non-Small Cell Lung Adenocarcinoma (H1299), oesophageal adenocarcinoma (FLO-1, OE-33).	GD-1: 239 nM	GC_50_: 52 kBq (PSN-1) and 89 kBq (AsPC-1)	ND	SPECT imaging only	[34]
11. [^123^I]CC1 MA = 18–342 GBq/µmol	Breast cancer (MD MB-231), pancreatic cancer (AsPC1, PSN1 and Capan1) and glioblastoma (U87MG)	CC1: 2.9 nM (PARP1) and 0.6 nM (PARP2)	631 ± 35 Bq/200µL	γH2AX increase	1 × 3 MBq (i.v.): delayed tumour growth and prolonged survival; Target blocking confirmed	[39]
12. [^125^I]KX-02-019 MA = ND	Ovarian cancer (SNU-251), EMT-6, MEF (PARP 1 or 2 KO (-/-))	13.9 ± 1.1 nM (Ki)	ND	ND	ND	[31]
13. [^125^I]KX1 MA = 81.4 GBq/µmol	Neuroblastoma (19 cell lines)	Not reported	12.5 ± 0.9 nCi/mL (IMR-05), 980 ± 60 nCi/mL (NLF)	γH2AX increase	SPECT imaging only	[12, 28, 29, 32]
14. Para-[^125^I]PARPi/ [^125^I]PARPi-01 MA = 30.52 ± 6.4 GBq/µmol	Triple-negative breast cancer (8 cell lines); Non-TNBC BRCA^wt^ cells: MCF7, SKBR3, mammary epithelial cell line (MCF10A)	Not reported	2.3 ± 0.43 MBq/ mL	γH2AX increase; Apoptosis increase (Annexin V; TUNEL)	4 × (8 ± 3 MBq per dose, every 10 days, i.v.): no significant survival benefit upon treatment; Target blocking not confirmed	[13, 30]
15. [^77^Br]Br-WC-DZ /[^77^Br]RD1 MA = 300 ± 120 GBq/µmol	Prostate cancer (PC3, IGR-CaP1), ovarian cancer (ID8, OVCAR8, OVCAR8-PARP1 KO, UWB1.289, and UWB1.289 + BRCA1)	ND	[^77^Br]Br-WC-DZ : 0.079 nM (PC3) and 0.41 nM (IGR-CaP1) [^77^Br]RD-1: 440 kBq/mL (ID8), 70 kBq/mL (OVCAR8), 200 kBq/mL (OVCAR8 PARP1^null^), 61 kBq/mL (UWB1.289). and 68 kBq/mL (UWB1.289 + BRCA1)	γH2AX and 53BP1 increase; Comet assay; G2/M cell cycle arrest	1 × 56 MBq (i.v.): prolonged survival up to 72 days; Target blocking confirmed	[40–42]

*ND* not determined

Radiolabelling strategies are closely linked to the radionuclide selection. Most reported studies employing radiohalogens utilise halogen-exchange approaches, such as copper-mediated iododeboronation of boronic pinacol ester precursors [39]. In contrast, radiometal-based approaches require the incorporation of chelators (e.g., DOTA), which can adversely affect the physicochemical properties of the ligand [25]. In particular, the increased polarity associated with chelator conjugation may hinder passive diffusion across cell membranes, thereby limiting efficient intracellular and nuclear accumulation.

In addition to these design considerations, PARP-TRT may offer intrinsic advantages as a DNA-targeting strategy compared with previously reported DNA-targeting radionuclide therapies. Iodine-125-labelled anthracyclines diffuse into cancer cells and intercalate directly into DNA, while radiohalogenated nucleoside analogues (e.g., ^123^I/^125^I/^131^I/^77^Br-labelled 5-iodo-2′-deoxyuridine [IUdR]) are incorporated into DNA via nucleoside transporters, as reviewed by Ku et al. [66]. However, these earlier approaches were associated with significant limitations, including the inability of DNA intercalators to distinguish between normal proliferating tissues and tumour cells, as well as rapid metabolic degradation, both of which contribute to toxicity. In contrast, PARP-TRT enables more selective targeting of DNA damage through PARP binding, supporting its continued development as a promising targeted therapeutic strategy.

### Toxicity aspects and route of administration of PARP-TRT

PARP1 is one of the most abundantly expressed nuclear proteins, reflecting its central role in DNA damage repair and maintaining genomic integrity. Although it is present in normal cells, PARP expression is generally higher in tumour cells, which may contribute to reducing toxicity. Tumour cells often exhibit elevated PARP1 levels due to an increase in DNA damage and replication stress, with up to approximately a million PARP1 molecules per cell, whereas normal cells express substantially lower levels [67]. Importantly, PARP1 activity and its association with (damaged) DNA are highly context-dependent, and are far higher in tumour compared to normal tissue, although this may vary significantly across different cancer cell lines [68].

The clinical translation of novel TRT agents must carefully consider potential toxicity, particularly off-target effects. As monotherapy with non-radiolabelled drugs, PARP inhibitors are associated with well-described toxicities, most notably haematological adverse effects such as anaemia. This is largely attributed to PARP trapping, which induces cytotoxicity in various organs with relatively high PARP expression, particularly those with high proliferative rates. Hepatic, gastrointestinal, and renal toxicities have also been reported [69].

In the context of PARP inhibitors as a radiosensitiser for EBRT and/or TRT, PARP inhibition in tumour tissue compared with normal tissue results in DNA damage repair inhibition, and increased radiocytotoxicity. A toxicity profile similar to that of PARPi monotherapy may be expected, in addition to exacerbated normal tissues toxicity where EBRT and TRT act [70, 71].

Relating to PARP-targeted TRT, to date, only a handful of PARP-TRT agents have reported potential toxicities, in preclinical studies. Following administration of para-[^123^I]PARPi, transient lymphocyte alterations were observed, including an initial increase in lymphocyte counts. This was accompanied by temporary elevations in biomarkers associated with hepatotoxicity and muscle damage; however, these effects returned to baseline within two weeks [37]. Similarly, toxicity evaluations of [^123^I]CC1 demonstrated limited toxicity in normal tissues despite accumulation in organs identified in the biodistribution profile, including the liver, intestines, and kidneys involved in clearance, and spleen. These findings suggest minimal radiotoxic effects of Auger electron emissions in these tissues [39]. Comparable findings were reported for para-[^125^I]PARPi, in which low absorbed doses were observed in clearance organs and the liver [30].

For alpha particle-emitting radionuclides, a study of [^211^At]Parthanatine evaluated administered activities up to 36 MBq/kg. Haematological toxicity assessment performed 72 h after the final dose fraction demonstrated dose-dependent lymphocyte depletion, consistent with the relatively high PARP1 expression observed in lymphocytes within the blood compartment, whereas neutrophil counts remained unaffected, due to their comparatively low PARP1 expression. Bone marrow toxicity of [^211^At]Parthanatine, evaluated via colony formation assays of early progenitor cells, was detected only at the highest dose tested (60 MBq/kg). Granulocyte and megakaryocyte progenitors showed greater sensitivity, with decreased colony formation across all dose levels; however, recovery occurred within 2 weeks, and full restoration was achieved 4 weeks after treatment at 36 MBq/kg. These findings indicate a dose-dependent but largely reversible haematological toxicity profile, supporting the importance of careful dose optimisation in PARP-targeted radionuclide therapy. These effects are consistent with known PARP1 expression in haematopoietic progenitor compartments and highlight their radiosensitivity, particularly to alpha-emitting radionuclides [21].

Overall, these toxicity evaluations suggest that Auger electron-emitting radionuclides may offer an additional therapeutic advantage for PARP-TRT. Their ultra-short penetration range enables highly localised DNA damage following PARP binding, potentially improving the targeting of micro-metastases in PARP-expressing cancers while limiting collateral toxicity to surrounding healthy tissues, where PARP may be less associated with DNA [38].

Administration strategies of PARP-TRT are varied. While most compounds were administered intravenously, p-[^123^I]PARPi and p-[^131^I]PARPi have also been administered intratumorally, intrathecally or via CED, aimed at improving tumour uptake [26, 38]. Fractionated treatment has also been reported in several studies, where a single high dose is divided into multiple fractions, as demonstrated for [^211^At]Parthanatine, meta-[^131^I]PARPi, and para-[^125^I]PARPi, where it was shown to reduce tumour burden and prevent tumour regrowth while potentially decreasing toxicity compared to single high dose administration. However, fractionated dosing strategies remain largely underexplored for other PARP-TRT compounds, which are predominantly administered as single-dose regimens, as summarised in Table 2 [13, 21, 26].

As several studies have focused on targeting glioblastoma, intravenous administration may face constraints due to the presence of the blood-brain barrier when it remains intact. However, intravenous injection can still result in significant tumour uptake in cases where the BBB is disrupted, such as in high-grade gliomas. Another alternative approach, such as intrathecal administration and CED, represents a viable alternative for delivering the compound, especially for targeting brain tumours. Nevertheless, many currently reported PARP-TRT agents and PARPi exhibit low solubility and poor blood-brain barrier penetration. The development of compounds capable of crossing the blood-brain barrier, therefore, represents a promising direction for PARP-TRT, as demonstrated previously for PARP imaging agents such as [^11^C]AZ3391 and [^18^F]AZD9574 [72, 73].

### Model selection and radiobiology of PARP-TRT

Preclinical models for PARP-TRT were mainly selected based on the PARP expression levels of the tumour models. In vitro studies using PARP1 or PARP2 KO cell lines provide an excellent control for the specific binding of PARP-TRT candidates to the target. However, a previous study reported that PARylation, even at very low levels of PARP1/2, is sufficient to recruit DNA repair enzymes [74].

A major limitation of the currently reported studies is the wide variety of models and assays used to evaluate PARP-TRT candidates. This makes direct comparison less straightforward. In several studies, we found that basic parameters were not reported. In particular, absolute in vivo uptake values were frequently absent, with some studies reporting only uptake ratios, which offer limited information regarding potential therapeutic efficacy. Moreover, tumour retention time, blocking studies and other selectivity evaluations were not described. Given that PARP is an exclusively nuclear protein, blocking studies may give some hint towards nuclear localisation of the compound. More specifically, confirmation of nuclear uptake by cell fractionation would provide direct evidence; however, this was not reported in most studies, despite offering important insights into the mechanism of action, dosimetry, and potential therapeutic response. This is especially poignant for the short-range Auger electron-emitting radiolabelled PARP inhibitors, where more internalisation in the nucleus, and closer proximity to tumour chromatin through interaction with trapped PARP would be more radiocytotoxic, as has been reported for, [^123^I]CC1, p-[^125^I]PARPi and p-[^123^I]PARPi [30, 36, 39]. Key parameters to report in preclinical TRT studies were recently summarised by the European Working Group of Radiobiology of Molecular Radionuclide Therapy and published in EJNMMI in 2025 [75].

**Fig. 4:**
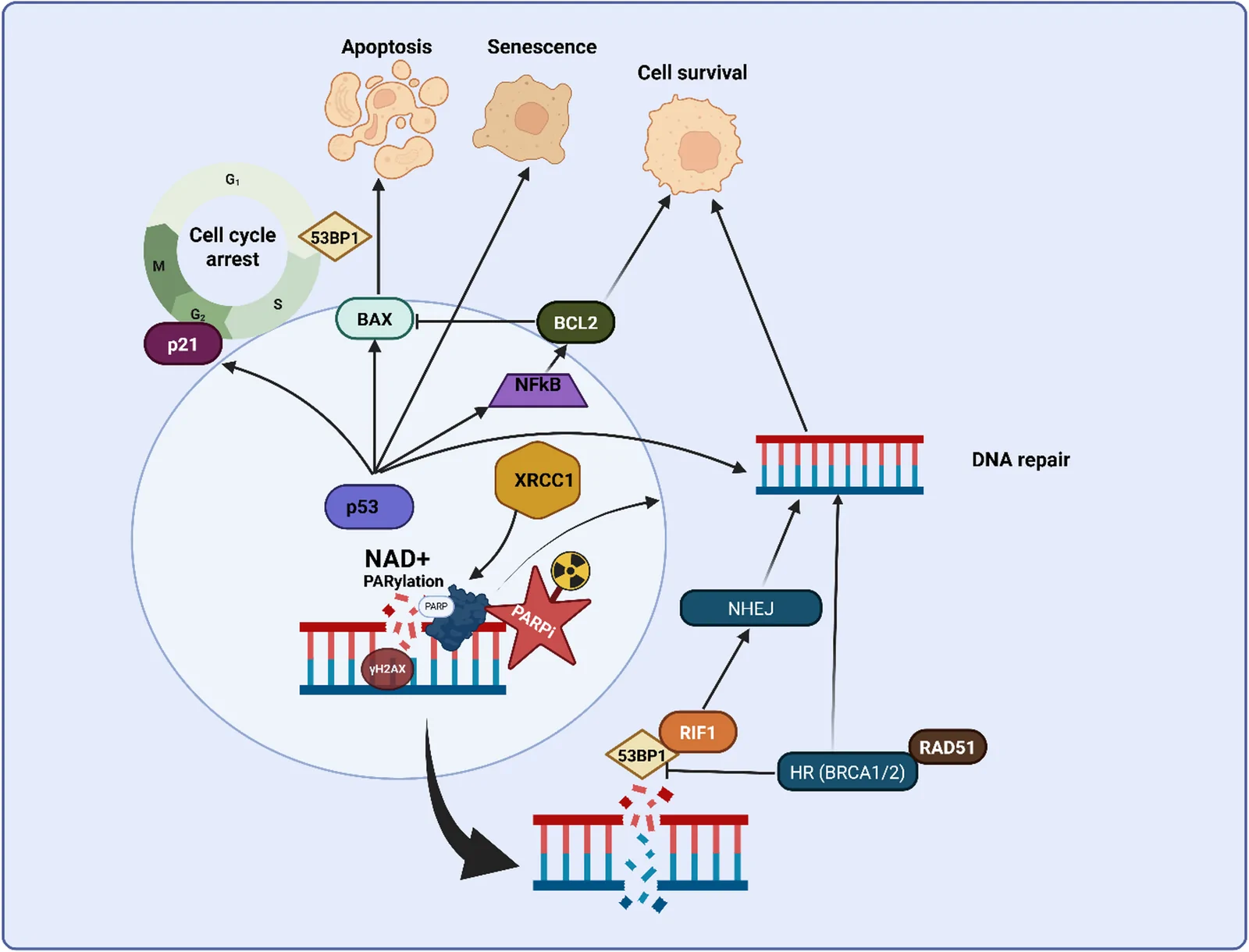
Integrated mechanism of DNA damage responses and potential biomarkers for PARP-TRT evaluation

Additionally, there is limited insight into the molecular and cellular mechanisms underlying (and predicting) treatment response, which hinders both a comprehensive understanding of therapeutic effects and the development of patient selection strategies. DNA damage response is activated upon the occurrence of DNA lesions, such as those induced by PARP-TRT, as shown in Fig. 4. The earliest response involves the activation of PARP enzymes. This modification facilitates the recruitment of DNA repair enzymes to the sites of damage [1]. Concurrently, phosphorylation of γH2AX occurs at DNA break sites, serving as a marker for double-strand breaks and as a platform for the assembly of additional repair proteins [76]. In line with this concept, Makvandi et al. demonstrated that [²¹¹At]Parthanatine treatment induces dose-dependent DNA damage in neuroblastoma cells, leading to upregulation of PARP1, consistent with activation of the DNA damage response pathway [22].

The tumour suppressor protein p53 plays a central role in orchestrating downstream signalling following DNA damage. Activated p53 induces the transcription of p21, a cyclin-dependent kinase inhibitor that promotes cell cycle arrest, particularly at the G1 and G2 checkpoints, allowing time for DNA repair or the initiation of cellular senescence [77]. Alternatively, p53 activation can upregulate pro-apoptotic factors such as BAX, leading to programmed cell death (apoptosis) [78]. In contrast, activation of NF-κB can promote cell survival by inducing BCL2 expression, thereby inhibiting apoptosis and facilitating DNA repair. Upon the formation of DNA double-strand breaks, 53BP1 is recruited to the damage site, where it mediates non-homologous end joining through its interaction with RIF1 [79]. As summarised in Table 2, biomarkers such as *γ*H2AX and 53BP1 have been the most frequently investigated in relation to PARP-TRT. In the context of PARP-TRT, studies using p-[^123^I]PARPi demonstrated preferential targeting of p53-mutant colorectal cancer models, resulting in greater DNA damage compared with p53–wild-type tumours [37]. This finding aligns with the classic lethality paradigm and highlights the potential relevance for assessing molecular pathways predominantly affected by PARP-TRT.

#### Dosimetry aspects

One important parameter for evaluating treatment efficacy is the radiation-absorbed dose of TRT, a metric of cytotoxic insult. The calculation for absorbed dose itself is not straightforward and is still not commonly performed among all TRT developments, especially for short-pathlength particle emitters [80]. Currently, microdosimetry for PARP-TRT is most commonly performed using MIRDcell, as best available methodology [81]. By incorporating uptake, efflux, and cellular fractionation data as inputs, this approach has been applied to several PARP-TRT agents, as shown in Table 3. Nevertheless, the key limitation remains, especially for Auger electron emitters, given the simplified non-realistic cell geometries, which can significantly affect calculated metrics. In addition, it may be challenging to calculate the true radiation absorbed dose to tumour DNA, because of the dynamic interaction of very short-range Auger electron-emitting PARP inhibitors [82]. S-value calculations often assume that PARP is confined within the nucleus; however, when radiolabelled PARP inhibitors localise specifically to chromatin, the resulting absorbed dose to DNA may be substantially higher. Therefore, radiocytotoxicity to tumour cells may be underestimated, but so may normal tissue toxicity.

**Table 3:** Reported dosimetry data for the current PARP targeted radionuclide therapy agent

Compound	Methods/ tools	In vitro results	In vivo results	Ref.
[^211^At]Parthanatine	OLINDA/EXM software based on time activity curves fitting from biodistribution data	ND	Tumour absorbed dose: 2.15 Gy/dose at 36 MBq/kg (NB-EBC1x tumours)	[21]
[^123^I]CC1	MIRDcell (uptake and retention data)	Absorbed radiation dose (24 h): approximately 8 Gy	ND	[39]
p-[^123^I]PARPi	PARaDIM v1.0	D_37_ U251 = 0.05 Gy	Absorbed dose : Tumour = ~ 8.0 cGy/MBq Lung = ~ 1.6 cGy/MBq Kidney = ~ 2.3 cGy/MBq	
[^77^Br]Br-WC-DZ	MIRDcell (cellular dosimetry) and OLINDA/EXM v1.1 (human organ dose; mouse %ID/g uptake-based extrapolation)	D_50_ OVCAR8 = 4.3 ± 0.3 Gy D_50_ OVCAR9 PARP1 KO = 3.4 ± 0.6 Gy	Absorbed dose : Heart = 8.2 ± 0.8 mGy/MBq Lung = 10.3 ± 0.7 mGy/MBq Kidney = 16 ± 4 mGy/MBq Effective dose: 16 ± 4 mSv/GBq	[42]
Meta- [^131^I]PARPi	Digimouse atlas used as a phantom; time-activity curves fitted using a 1-phase exponential decay model	ND	Calculated absorbed dose: 134.1 cGy/MBq Thyroid: 68.6 cGy/MBq	[26, 83]
[^125^I]KX1	MIRDcell V2.1	D_50_ UWB1.289 2.31 ± 0.21 Gy and UWB1.289-BRCA1 restored 3.45 ± 0.36 Gy	ND	[12, 29]
[^177^Lu]Lu-DOTA-PEG-PARPi	OLINDA/EXM 2.2.3 (pharmacokinetic input data)	ND	Absorbed dose : Spleen = 5.52 × 10^− 3^ mGy/MBq Red marrow = 4.36 × 10^− 3^ mGy/MBq Lung = 2.33 × 10^− 3^ mGy/MBq Kidneys = 2.00 × 10^− 3^ mGy/MBq Effective dose: 2.00 × 10^− 2^ mSv/MBq	[25]

D_50_ = Radiation dose for 50% survival, D_37_ = Radiation dose for 37% survival, *ND* Not determined

Furthermore, clinically available data, e.g. from PET or SPECT imaging, produce quantitative information on a mm-to-cm scale, limited by the respective resolution of the imaging technique. However, it does not provide information on the microscopic distribution of the Auger-electron-emitting PARP inhibitor within a cell. This necessitates other modelling approaches during translation to ensure safety.

### Combination therapy

To improve the treatment efficacy of TRT, several combination strategies have been investigated, particularly those that enhance DNA damage. One such approach involves inhibition of DNA repair pathways. For example, the DNA-dependent protein kinase catalytic subunit (DNA-PKcs) inhibitor AZD7648 has been combined with [^177^Lu]Lu-DOTA-TATE, resulting in enhanced therapeutic efficacy both in vitro and in vivo models [84]. Similarly, PARP inhibitors have been widely investigated as radiosensitizers due to their ability to potentiate the effects of ionising radiation. The radiosensitisation induced by PARP inhibitors is mediated through multiple mechanisms, including inhibition of DNA repair, replication-dependent radiosensitisation, modulation of chromatin remodelling, reversal of hypoxia-induced radioresistance, and induction of G2/M cell cycle arrest. Recent preclinical and clinical studies have demonstrated that PARP inhibitors are promising radiosensitising agents [54].

Although most studies have been conducted in the context of EBRT, they provide a strong rationale for their application in targeted radionuclide therapy (TRT). For instance, the combination of PARP inhibitors with other chemotherapeutic agents is currently under investigation in clinical trials, as reviewed by Chan et al. In addition, olaparib has been evaluated as a radiosensitiser in pancreatic ductal adenocarcinoma (PDAC) mouse models, while talazoparib has demonstrated radiosensitising effects in small-cell lung cancer cell lines [85]. In the context of PARP-TRT, certainly, the very low concentrations needed for PARP-TRT do not result in pharmacologically relevant inhibition of PARP. Global PARP inhibition is therefore unlikely to play a role. However, whether PARP inhibitors can be effectively combined with PARP-TRT remains an open question. A likely limitation is competitive binding, whereby non-radiolabeled PARP inhibitors may interfere with the tumour uptake of radiolabelled PARP ligands. In addition to radiosensitisation, other combination strategies aim to enhance PARP-TRT efficacy by increasing target availability. For example, pretreatment with doxorubicin-loaded nanoparticles has been shown to upregulate PARP expression and enhance nuclear uptake of p-[^125^I]PARPi [30]. Nevertheless, combining chemotherapy with PARP-TRT requires careful evaluation to determine whether the interaction is synergistic, additive, or antagonistic. Optimal dosing schedules must be established, and the toxicity profile should be systematically compared with that of each monotherapy. PARP-TRT has also shown potential as an immunomodulatory strategy. Treatment with [^211^At]Parthanatine has been reported to enhance CD4⁺ T-cell–mediated immune surveillance within the tumour microenvironment and to improve tumour growth inhibition when combined with anti–PD-1 therapy, compared with either treatment alone [20].

Previous work with [^211^At]Parthanatine in neuroblastoma xenografts demonstrated that sublethal administration of PARP-TRT induces DNA damage accompanied by a transient upregulation of PARP1 expression in tumour cells, with peak levels observed 6–24 h post-treatment, depending on dose. This increase was associated with enhanced uptake of the PARP1 targeted PET tracer [^18^F]FTT, indicating that therapy-induced DNA damage may transiently increase target availability for subsequent PARP-targeted interventions [24]. While these findings support the concept of exploiting DNA damage to modulate PARP1 expression, approaches aimed at deliberately inducing such upregulation using external DNA-damaging modalities, such as EBRT, remain to be investigated.

### Future prospects and conclusions

Radionuclide selection is a critical determinant of efficacy in PARP-TRT. Based on current reports, Auger electron emitters, including ^123^I, ^125^I, and ^77^Br, offer great potential due to their high linear energy transfer and subcellular-range radiation, which enable selective DNA damage within the nucleus of cancer cells. Alternative strategies using radiohalogen β- or α-emitters face limitations such as high normal tissue burden and off-target toxicity, whereas radiometal-labelled PARP inhibitors face limitations that may restrict nuclear access, currently constraining their therapeutic suitability.

Preclinical studies of radiolabeled PARP inhibitors have demonstrated robust target engagement and induction of DNA damage. Several agents exhibit selective tumour uptake and activation of DNA damage pathways, providing a strong rationale for further development. However, progress has been hindered by heterogeneous preclinical models and inconsistent reporting, which complicate direct comparisons across studies. Critical gaps remain in the standardisation of assays to assess selectivity and subcellular localisation, both of which are essential for optimising nuclear targeting and radiocytotoxicity. Comprehensive evaluation of tumour-specific biomarkers, detailed radiobiological characterisation, and integration of these data for patient stratification are also needed to fully define the therapeutic potential of PARP-TRT.

Besides that, treatment selection for PARP-TRT using PARP-targeted SPECT/PET imaging agents, such as [^18^F]FTT, [^18^F]PARPi, and [^18^F]olaparib, remains largely underexplored. Although [^18^F]FTT has been proposed as a theranostic imaging counterpart for the alpha-emitting candidate [^211^At]Parthanatine, clinical validation remains limited. Other PARP-TRT candidates emitting Auger electrons may provide additional advantages, particularly when paired with SPECT imaging agents such as [^123^I]CC1 or p-[^123^I]PARPi, both of which have demonstrated utility for detecting PARP-expressing tumours. PARP-targeted imaging using SPECT or PET may therefore represent a useful, although not essential, strategy for patient selection prior to PARP-TRT, as high tracer uptake could correlate with improved therapeutic response. Nevertheless, low tumour uptake does not necessarily predict poor therapeutic efficacy, since strong DNA association of the radiolabelled PARP inhibitor may still result in effective tumour cell killing. To date, no predictive biomarkers for response to PARP-TRT have been established.

In conclusion, systematic preclinical and translational studies to address these gaps are essential for defining the clinical utility of PARP-TRT. Continued investigation of radiolabelled PARP inhibitors, followed by standardised methodologies and biomarker-guided strategies, will advance precision radionuclide therapy and may expand its role in personalised cancer treatment.

## Data Availability

Not applicable.
